# Genetic variability in Brazilian *Capsicum baccatum* germplasm collection assessed by morphological fruit traits and AFLP markers

**DOI:** 10.1371/journal.pone.0196468

**Published:** 2018-05-14

**Authors:** Rafaella Cardoso, Claudete F. Ruas, Renata M. Giacomin, Paulo M. Ruas, Eduardo A. Ruas, Rosa L. Barbieri, Rosana Rodrigues, Leandro S. A. Gonçalves

**Affiliations:** 1 Biology Department, Universidade Estadual de Londrina, Londrina, Paraná, Brazil; 2 Embrapa Clima Temperado, Pelotas, Rio Grande do Sul, Brazil; 3 Plant Breeding Department, Universidade Estadual do Norte Fluminense *Darcy Ribeiro*, Campos dos Goytacazes, Rio de Janeiro, Brazil; 4 Agronomy Department, Universidade Estadual de Londrina, Londrina, Paraná, Brazil; National Cheng Kung University, TAIWAN

## Abstract

*Capsicum baccatum* is one of the main pepper species grown and consumed in South America. In Brazil, it is commonly cultivated by family farmers, using mostly the genotypes bishop's hat genotypes (locally *cambuci*) and red chili pepper (*dedo-de-moça*). This study had the objective of characterizing 116 *C*. *baccatum* accessions from different regions of Brazil, based on morphological fruit descriptors and AFLP (Amplified Fragment Length Polymorphisms) markers. Broad phenotypic variability among the *C*. *baccatum* accessions was detected when using morphological fruit descriptors. The Ward modified location model (Ward-MLM) discriminated five groups, based mainly on fruit shape. Six combinations of AFLP primers detected polymorphism in 97.93% of the 2466 identified bands, indicating the high genetic variability in the accessions. The UPGMA coincided with the Bayesian clustering analysis and three large groups were formed, separating the wild variety *C*. *baccatum* var. *praetermissum* from the other accessions. There was no relation between genetic distance and geographical origin of the accessions, probably due to the intense exchange of fruits and seeds between farmers. Morphological descriptors used together with AFLP markers proved efficient in detecting the levels of genetic variability among the accessions maintained in the germplasm collections. These results can be used as an additional source of helpful information to be exploited in *C*. *baccatum* breeding programs.

## Introduction

The genus *Capsicum* (Solanaceae) is native to the tropical zones of Central and South America, and was one of the first genera to be domesticated, around 6000 B.C. [1]. The genus comprises approximately 38 described species, with great morphological variation, mainly in terms of fruit color, size, shape and levels of pungency [2]. Among these species, five are considered domesticated: *C*. *annuum* L., *C*. *chinense* Jacq., C. *frutescens* L., *C*. *pubescens* Ruiz et Pav., and *C*. *baccatum* L. (var. *pendulum*) [3].

Among the domesticated peppers species, *C*. *baccatum* is one of the most important in South America, cultivated mainly in plains and medium elevations of Argentina, Bolivia, Peru, Ecuador, Paraguay, Colombia, Chile, and in many different biomes in Brazil [4]. The center of origin of this species is Bolivia and Southern Peru [5,6], and it is divided into four botanical varieties: the domesticated *C*. *baccatum* var. *pendulum* and wild *C*. *baccatum* var. *baccatum*, *C*. *baccatum* var. *praetermissum*, and *C*. *baccatum* var. *umbilicatum* [5,7].

In Brazil, landraces of *C*. *baccatum* var. *pendulum* are widely cultivated by family farmers, where the bishop's hat (locally named as *cambuci*) and the red chili (*dedo-de-moça*) peppers are the most commonly grown and used in Brazilian gastronomy, consumed fresh or in processed form [8,9,10]. The red chili type or hot pepper, named in Brazil as *chifre de veado* or *dedo-de-moça*, characterized by its elongated fruits, is widely consumed in the South and Southeast Brazilian regions, where it is used for sauces or in the form of dehydrated flakes, with mild to median pungency. The *cambuci* or bishop’s hat (*chapéu de bispo*) type has bell-shaped fruits with mild to absent pungency, and can be consumed fresh, in salads, cooked or canned, mainly in the Southeastern region of Brazil [11].

The conservation of this diversity in genebanks is imperative, in view of the genetic erosion caused by the destruction of natural habitats, substitution of local varieties by improved cultivars, and the agricultural abandonment of pepper-producing small holders [12]. However, the genebanks installed as genetic repositories are often affected by mismanagement, consisting of a series of mistakes, such as inadequate storage, duplicate accessions and loss of variability due to genetic erosion. In this sense, the characterization of the accessions conserved in genebanks is understood as essential for the species’ conservation and use in plant breeding programs.

The characterization of a germplasm is commonly based on phenotypic descriptors and molecular markers [13]. Using amplified fragment length polymorphism (AFLP) markers, Albrecht et al [4], observed a greater genetic diversity in the wild accessions of *C*. *baccatum* var. *baccatum* when compared to *C*. *baccatum* var. *pendulum*. In addition, the *C*. *baccatum* var. *pendulum* accessions were divided in two genetic groups, one of the western region (Peru, Colombia, Ecuador, Bolivia, Chile, and western Argentina) and the other representing the eastern region (Brazil, Paraguay, and eastern Argentina), suggesting multiple sites of domestication. In accessions from Brazil, [10,14,15] observed wide phenotypic and genotypic variability among accessions of *C*. *baccatum* var. *pendulum*, and distinct dissimilarity patterns between phenotypic and genotypic data, indicating that both (morphoagronomic and molecular) characterization stages are fundamental for a more detailed knowledge and better discrimination of *C*. *baccatum* accessions [10].

The objective of this study was to characterize *C*. *baccatum* accessions from different germplasm collections and different regions of Brazil by means of morphological fruit descriptors and AFLP molecular markers. The results of this study will increase the knowledge about the variability found in *C*. *baccatum* accessions, contributing to the conservation of the species, as well as the identification of potential accessions to be used in breeding programs.

## Material and methods

### Phenotyping: Morphological characterization of fruits

**Fig 1:**
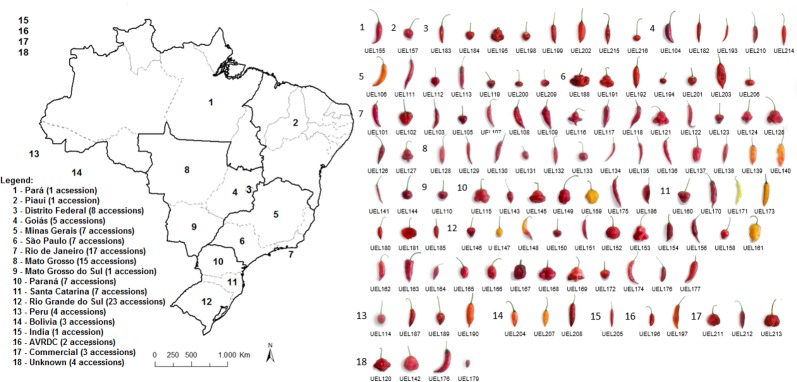
Geographic distribution of 116 *Capsicum baccatum* accessions in Brazil of genebank of Universidade Estadual de Londrina (UEL).

A total of 116 *C*. *baccatum* accessions from 12 Brazilian states in the five geographic regions of Brazil and representative accessions from Peru (4), Bolivia (3), India (1) and two from the Asian Vegetable Research and Development Centre (AVRDC) were evaluated (Fig 1). The accessions were obtained from the genebank of the Empresa Brasileira de Pesquisa Agropecuária (Embrapa–Clima Temperado and Embrapa Hortaliças) and from the Universidade Estadual do Norte Fluminense *Darcy Ribeiro* (UENF), forming the genebank of the Universidade Estadual de Londrina (UEL).

For the experiments, the accessions were sown in 128-cell polystyrene trays containing the substrate Vivatto®. After 30 days, the seedlings were planted in a protected cultivation area of UEL, Londrina, Paraná, Brazil. The fruit morphology was characterized based on the descriptors proposed by the International Plant Genetic Resources Institute (currently named Biodiversity International) [16]. Fourteen fruit descriptors were used, of which 10 were qualitative: anthocyanin spots or stripes, fruit color at intermediate stage, fruit color at mature stage, fruit shape, fruit shape at pedicel attachment, fruit blossom end appendage, fruit shape at blossom end, fruit cross-sectional corrugation, number of locules per fruit and fruit surface; and four quantitative variables: fruit length, fruit width, fresh fruit weight, and fruit wall thickness.

### Genotyping: AFLP markers

For molecular analysis, the DNA was extracted separately from young leaves of five plants per accession. The samples were prepared using an automatic DNA extractor (Retch MM 400), followed by extraction as proposed by Doyle and Doyle [17] CTAB (Cetyltrimethylammonium Bromide, Sigma-Aldrich, Missouri-USA) method, except that CTAB in the extraction buffer was replaced by MATAB (Alkyltrimethylammonium Bromide, Sigma-Aldrich, Missouri-USA). The DNA quality and integrity were assessed by electrophoresis on 1% agarose gel. The DNA concentration was determined by a Nano drop Spectrophotometer 2000/2000c (Thermo Scientific, California-USA).

The AFLP technique was applied according to the protocol proposed by Vos et al [18], with modifications. The DNA extracted from five plants per accession was mixed proportionally. Approximately 700 ng of this DNA was double-digested with 1 U of MseI and 5 U of EcoRI (Thermo Scientific, California-USA) and ligated to the adapters EcoRI (0.5 μM) and MseI (5 μM) in a reaction containing: T4 DNA ligase (2U); buffer T4 DNA ligase 1X; NaCl (0.05 M); BSA (50 μg/μL); and DTT (0.25 mM) up to a final volume of 10 μL. The program for the digestion-ligation consisted of: 37°C for 4 h, 22°C for 1 h and 70°C for 10 min. The pattern of digestion-ligation products was visualized on 1% agarose gel. Once the digestion was confirmed, the amplified product was diluted 1:4 with ultrapure water.

Pre-selective amplification was performed using 3.5 μL of GoTaq® Green Master Mix (Promega, Winchester-USA), 0.58 μL of the pre-selective primers *Eco*RI+A and *Mse*I+C (4.75 μM) pre-selective primers, 3.0 μL of the dilution of the restriction-ligation mixture and ultra pure water to a volume of 10 μL. Pre-selective amplification consisted of: 1 cycle at 72°C for 2 min, 20 cycles at 94°C for 1 sec, 56°C for 30 sec, 72°C for 2 min and a final cycle at 60°C for 30 min. Pre-selective PCR amplification was confirmed on 2% agarose gel and the amplified product was diluted to 1:8 in ultrapure water. For the selective amplification, 12 combinations of selective *Eco*RI/*Mse*I primers were initially screened for polymorphism and repeatability. The six most polymorphic combinations were chosen for fluorescent labeling (*Eco*RI(FAM)/-ACA/*Mse*I-CAC, *Eco*RI(NED)-AGC/*Mse*I-CTGA, *Eco*RI(VIC)-ACT/*Mse*I-CAA, *Eco*RI(PET)-AGC/*Mse*I-CAG, *Eco*RI(VIC)-ACT/*Mse*I-CAG, and *Eco*RI(NED)-ACG/*Mse*I-CTGA) and selective amplification.

The selective reactions were carried out in a volume of 10 μL containing: 3.5 μL PCR Master Mix (GoTaq Green Master Mix, Promega, Winchester-USA), 0.54 μL of each primer *Mse*I (5 μM) and *Eco*RI (1μM), 2.5 μL of the diluted pre-amplification reaction mixture and 2.92 μL ultrapure water. The amplification program consisted of 1 cycle at 94°C for 2 min, 65°C for 30 sec and 72°C for 2 min; 8 cycles at 94°C for 1 sec, 64°C for 30 sec and 72°C for 2 min; 23 cycles at 94°C for 1 sec, 56°C for 30 sec and 72°C for 2 min and 1 final cycle at 60°C for 30 min. The fragments were resolved by capillary electrophoresis using the automated DNA analyzer model 3500xL (Applied Biosystems, California-USA). The results of the electrophoresed fragments were combined into a binary matrix by GeneMapper® software v.4.1 (Applied Biosystems).

### Data analysis

Phenotypic descriptors (qualitative and quantitative) were analyzed using the Ward-MLM (Modified Location Model) method, as proposed by Franco et al. [19], which allows the simultaneous analysis of qualitative and quantitative traits. For the clustering formation between accessions, the CLUSTER and IML procedures, available in the SAS program [20], were used. The distance matrix was determined by the Gower algorithm [21], for Ward's clustering. The ideal number of groups was defined based on the criterion of the likelihood function, maximized according to the MLM method [20]. The differences between groups were analyzed by canonical variables and Mahalanobis’ distance [20].

To analyze the molecular data, Jaccard’s distance matrix was calculated and later UPGMA (Unweighted pair-group method using arithmetic averages). Data were analyzed by software FAMD, v. 2.3 [22] and FigTree 1.4.3. Based on the molecular data, Bayesian clustering was also performed using Structure software v. 2.3.4 [23], based on the method described by Evano et al. [24], with 100,000 iterations (Monte Carlo Markov Chain), with a burn-in of 10,000 iterations, in a model assuming mixed clusters (admixture) and correlated allele frequencies. The cluster number was determined according to the method described by Evanno et al. [24], and the graphs were generated by the online interface Structure Harvester [25]. For the analysis of molecular variance (AMOVA) among accessions of the three genebanks software Arlequin 3.5 we used [26].

## Results

### Morphological characterization of fruits

Wide morphological variability was observed among the accessions (Fig 1). Six different colors were observed in the mature fruit stage. The red color was predominant, with a total of 97 accessions, followed by orange (8), dark red (5), orange-yellow (4), lemon yellow (1) and pale orange (1) colors (Table 1). In the intermediate fruit stage, 60 accessions were green, followed by 53 orange and 3 yellow accessions. All fruit shapes proposed by IPGRI [16] were observed, but elongated shape was predominant, with 46 accessions, followed by triangular, campanulate, almost rounded, and blocky shapes, with 35, 19, 10, and 6 accessions, respectively.

**Table 1:** Variables and accessions number per group for categorical traits in each of the five groups (G1, G2, G3, G4 e G5) formed by the Ward-MLM strategy from 116 *Capsicum baccatum* accessions.

Variable	Groups				
	G1 (18)	G2 (20)	G3 (37)	G4 (17)	G5 (24)
**Fruit color at intermediate stage**					
Yellow	-	-	1	1	1
Green	2	12	16	13	17
Orange	16	8	20	3	6
**Fruit color at mature stage**					
Lemon-yellow	-	-	-	-	1
Orange-yellow	-	1	1	1	1
Pale orange	-	-	-	-	1
Orange	1	-	2	1	4
Red	15	18	33	14	17
Dark red	2	1	1	1	-
**Fruit shape**					
Elongate	1	-	29	1	15
Almost round	5	-	-	4	1
Triangular	10	2	8	7	8
Campanulate	2	17	-	-	-
Blocky	-	1	-	5	-
**Fruit Surface**					
Smooth	13	8	17	6	8
Semiwrinkled	-	4	13	2	7
Wrinkled	-	-	6	1	7
Other (Smooth with stretch)	5	8	1	9	2
**Fruit shape at pedicel attachment**					
Acute	-	-	6	-	2
Obtuse	-	-	28	1	19
Truncate	8	13	3	13	3
Cordate	10	5	-	2	-
Lobate	-	2	-	1	-
**Fruit shape at blossom end**					
Pointed	1	-	28	3	20
Blunt	2	-	5	4	4
Sunken	13	5	4	8	-
Sunken and pointed	1	12	-	2	-
Other (specify)	1	3	-	-	-
**Fruit blossom end appendage**					
Absent	17	19	28	2	12
Present	1	1	9	15	12
**Anthocyanin spots or stripes**					
Absent	9	10	18	13	5
Present	9	10	19	4	19
**Fruit cross-sectional corrugation**					
Slightly corrugated	12	1	17	5	7
Intermediate	3	2	17	7	11
Corrugated	3	17	3	5	6
**Number of locules**					
One	-	-	-	1	-
Two	8	2	10	5	9
Three	10	11	23	10	11
Four	-	7	4	1	4

The fruit surface was predominantly smooth, and all classes of fruit shape at pedicel attachment were represented, with prevalence of the obtuse shape. Fruit shape at blossom end was mostly pointed, followed by sunken, sunken and pointed, blunt and other. Most of the accessions had no appendage at the fruit tip, and had anthocyanin spots in the immature stage. The fruit cross-sectional corrugation varied from slightly corrugated to intermediate corrugated and corrugated, and the number of locules varied from one to four, with predominance of three locules.

**Fig 2:**
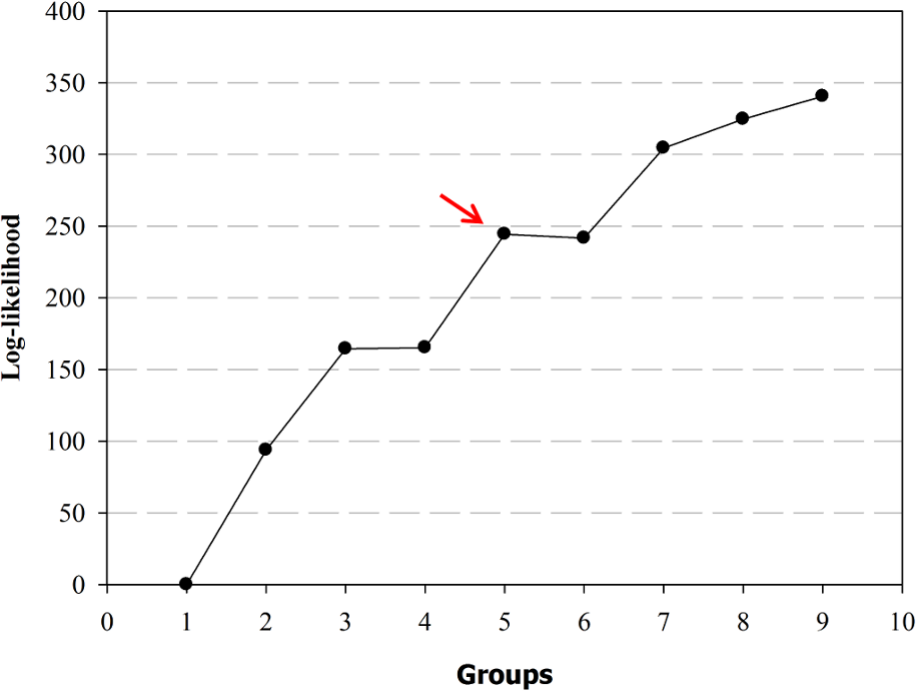
Boxplot for four fruits agronomic traits of 116 *Capsicum baccatum* accessions, for the five groups (G1-G5) formed by Ward’s hierarchical clustering analysis.

For the quantitative fruit descriptors, wide variability among accessions was also observed (Fig 2). Fruit length varied from 0.93 to 13.64 ($\bar{X}$ = 5.38 cm), while the width ranged from 0.40 to 5.90 cm ($\bar{X}$ = 2.48 cm). For fruit weight and wall thickness, the variation was 0.33 to 34.21 cm ($\bar{X}$ = 10.62 cm) and 0.01 to 0.46 cm ($\bar{X}$ = 0.21 cm), respectively.

**Fig 3:**
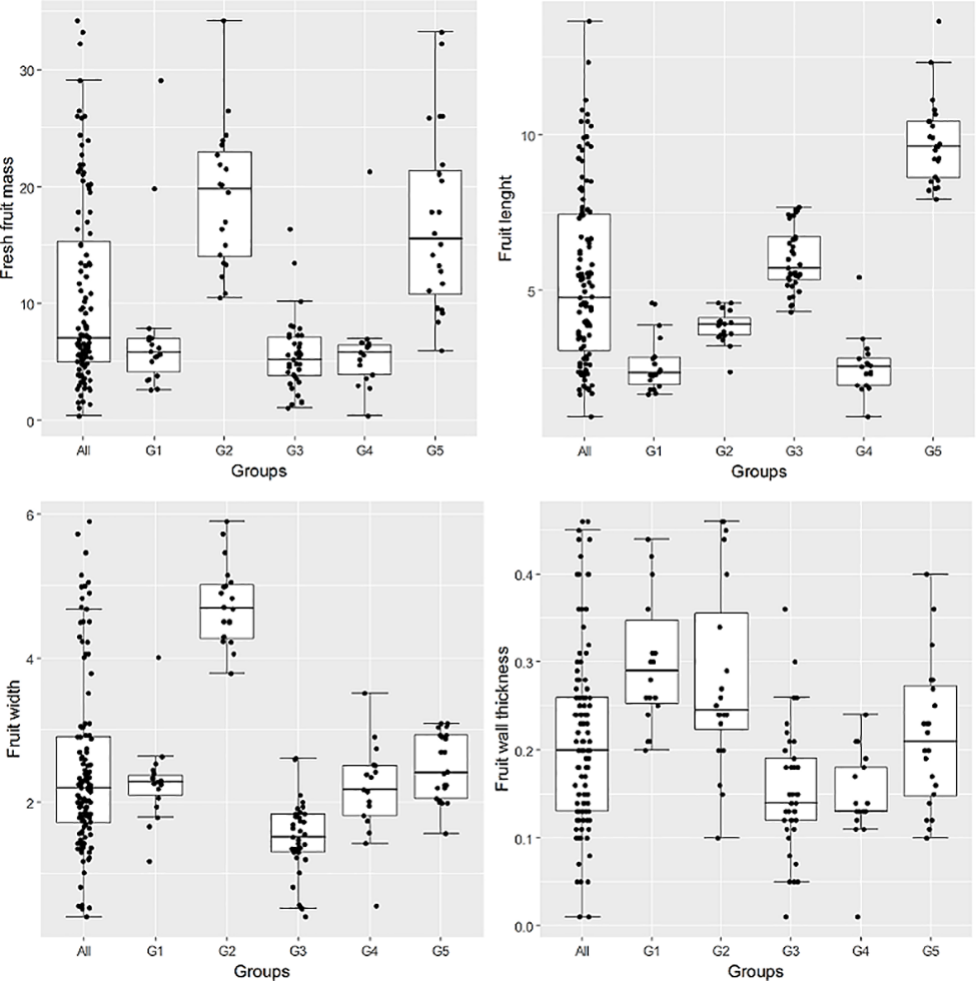
Graph of the logarithmic function of probability function (log-likelihood) showing the optimum number of groups for 116 *C*. *baccatum* accessions, characterized using morphoagronomic descriptors.

The Log-Likelihood function showed that the optimal number of groups was two or five, since the highest values were reached at these points (93.80 and 79.13, respectively) (Table 2; Fig 3). Group G1 consisted of 18 accessions, in which orange and red fruits predominated in the immature and mature stages, respectively, together with triangular shape and smooth surface (Table 1). Group G2, with 20 accessions, had fruits with mostly green and red color in the immature and mature stages, respectively, campanulate shape, with absence of appendage at the fruit tip. Group G3 associated 37 accessions, with prevalence of red fruits in the mature stage, and elongated fruit shape, while in group G4, 17 accessions were grouped, with predominantly green and red fruits in the immature and mature stages, respectively. Group G5, with 24 accessions, had mostly green and red fruits in the immature and mature stages, respectively, elongate fruits with obtuse shape at pedicel attachment and pointed shape at blossom end.

**Table 2:** Number of groups formed by the Ward-MLM strategy, based on the logarithmic probability function (log-likelihood) and its increment.

Number of groups	Log-likelihood	Increase
1	-1208.02	0.00
2	-1114.22	93.80*
3	-1043.64	70.58
4	-1042.80	0.83
5	-963.67	79.13*
6	-966.40	-2.73
7	-903.58	62.81
8	-883.33	20.25
9	-867.51	15.83

* Greatest increase

G2 and G5 clustered the heaviest fruits, and fruits in G2 were majority campanulate, had the largest width (Fig 3). For fruit length, the longest fruits (9.75 cm) were grouped in G5, while G1 and G4 clustered the lowest values (2.57 and 2.82 cm, respectively). For the variable wall thickness, the highest values were obtained for groups G1 and G2 (0.3 and 0.25 cm, respectively).

**Fig 4:**
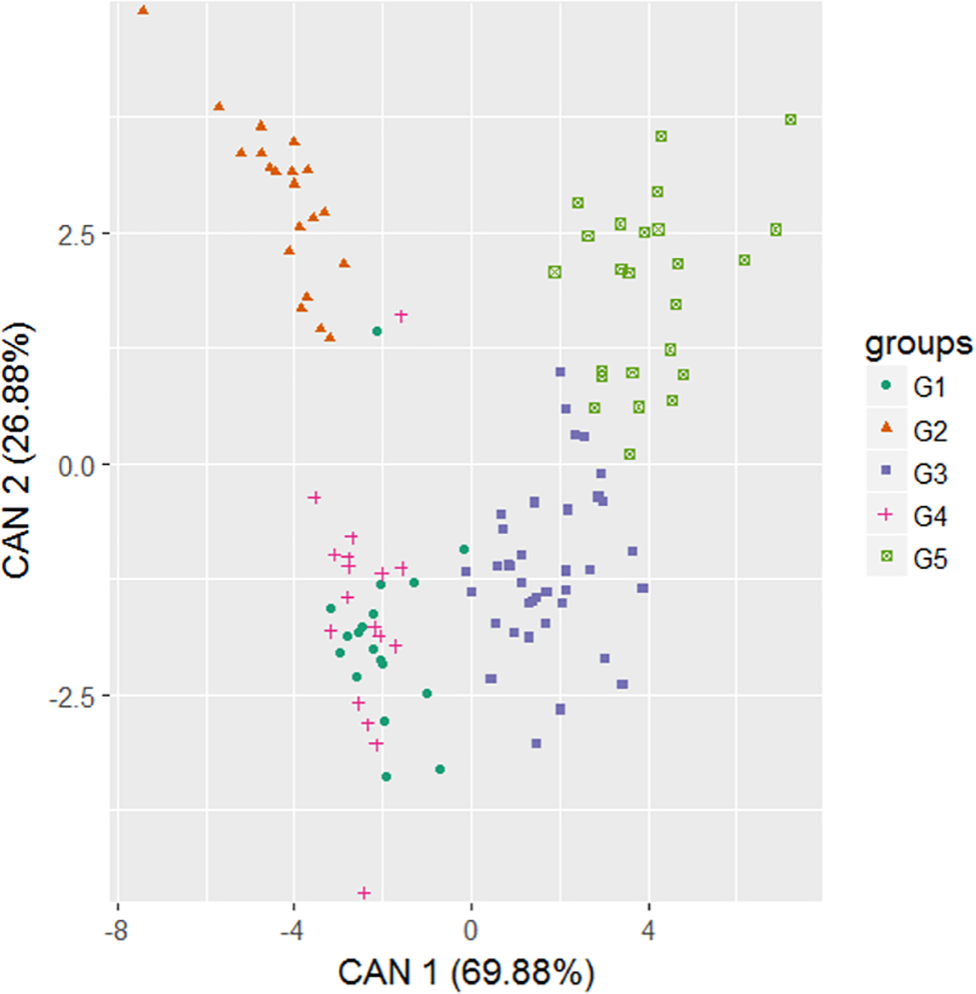
Dispersion of the first two canonical variables (CAN) with formation of five groups (G1–G5) by the Ward-MLM strategy, considering 116 *Capsicum baccatum* accessions.

The analysis of canonical variables (CAN) showed that the first two variables explained 96.76% of the total variation (CAN 1 and 2 with 79.88 and 26.88%, respectively) (Fig 4). The groups G2, G3 and G5 were allocated separately, while groups G1 and G4 overlapped, and had the smallest genetic distance (4.63) (Table 3). Groups G2 and G5 were the most distant from each other (69.42).

**Table 3:** Separation of groups by the Ward-MLM strategy based on Mahalanobis distance.

Groups	G1	G2	G3	G4
G2	28.63	0		
G3	17.13	51.77	0	
G4	**4,63**	23.60	18.34	0
G5	51.65	**69.42**	14.73	55.00

### Molecular characterization

Six selective AFLP primer combinations generated a total of 2466 fragments, distributed between 50 and 500 bp, of which 2415 (97.93%) were polymorphic. The number of polymorphic fragments ranged from 206 (*Eco*RI(FAM)-ACA/*Mse*I-CAC) to 545 (*Eco*RI(PET)-AGC/*Mse*I-CAG), and the combination *Eco*RI(FAM)-ACA/*Mse*I-CAC was proportionally the most polymorphic (99.51%).

**Fig 5:**
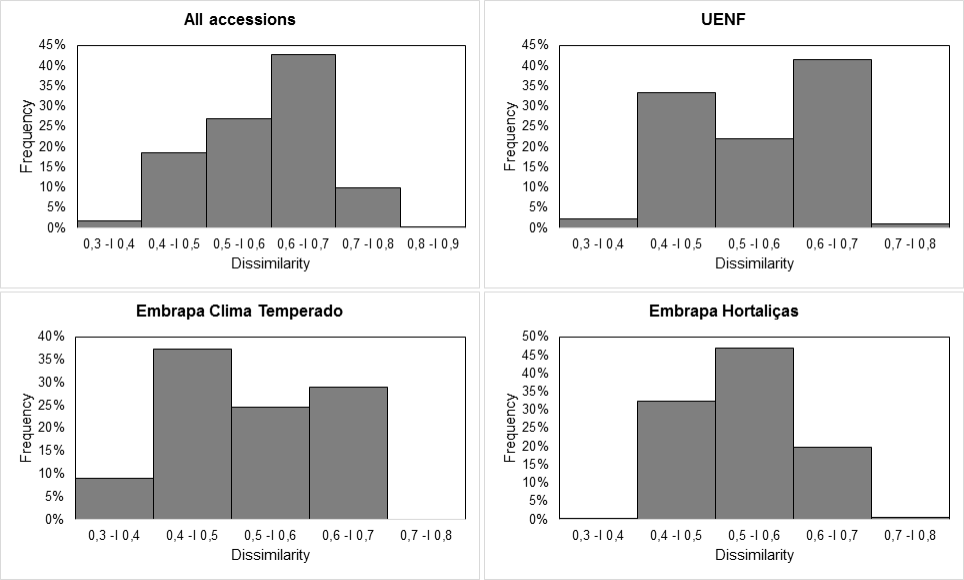
Frequency distribution of the dissimilarity based on AFLP markers among the 116 *C*. *baccatum* accessions.

The mean genetic distance, estimated by the Jaccard coefficient among all accessions, was 0.60. The classes with a genetic distance between 0.6 —| 0.7 and 0.5 —| 0.6 had the highest frequency (42.77 and 26.95%, respectively) (Fig 5). The genetic distance was smallest (0.32) between accessions UEL149 and UEL153 and greatest (0.8) between UEL182 and UEL105. When analyzing samples of the three gene banks separately, we found a mean distance of 0.55, 0.52 and 0.54, among the accessions of the collections from UENF, Embrapa Clima Temperado and Embrapa Hortaliças, respectively. The distribution of the frequency classes was broad in all three genebanks banks (Fig 5).

**Fig 6:**
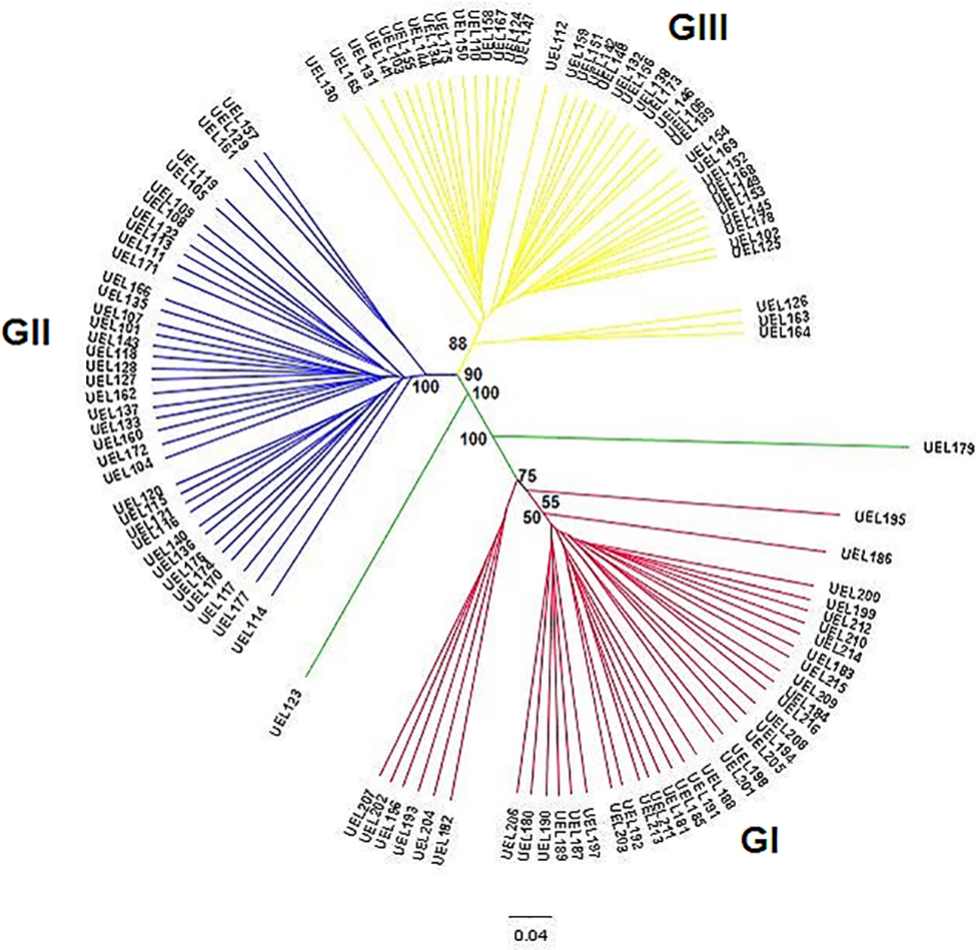
Dendrogram demonstrating genetic relatedness of 116 *Capsicum baccatum* accessions. Cluster analysis conducted using UPGMA with Jaccard—derived pairwise genetic distances.

The dendrogram, obtained by UPGMA hierarchical clustering analysis, identified the formation of three large groups, with a clear separation of the accessions UEL179 and UEL123, which can be considered outlier (Fig 6). Group I consisted of 37 accessions, all from the genebank of Embrapa Hortaliças, originally from the states of Goiás, Distrito Federal, São Paulo, Santa Catarina, Paraná, Minas Gerais, and representative accessions from Peru (UEL187, UEL189 and UEL190), Bolívia (UEL204, UEL207 and UEL208), India (UEL205) and AVDRC (UEL196 and UEL197), plus three commercial cultivars (Topseed (UEL211 and UEL212) and Agroflora-Sakata (UEL213). Group II, consisted of 37 accessions, including accessions from the UENF and Embrapa Clima Temperado gene banks, which were originally from the states of Rio de Janeiro, Rio Grande do Sul, Paraná, Santa Catarina, Pará, Mato Grosso, Minas Gerais and one accession from Peru (UEL114). Group III, consisting of 40 accessions, included accessions from the UENF and Embrapa Clima Temperado genebanks. Samples from group III were derived originally from the states of Mato Grosso, Rio Grande do Sul, Rio de Janeiro, Mato Grosso do Sul, Minas Gerais, Piauí, Santa Catarina, Goiás, and Paraná. There were no correlations between the distance matrices of the morphoagronomic and molecular data.

**Fig 7:**
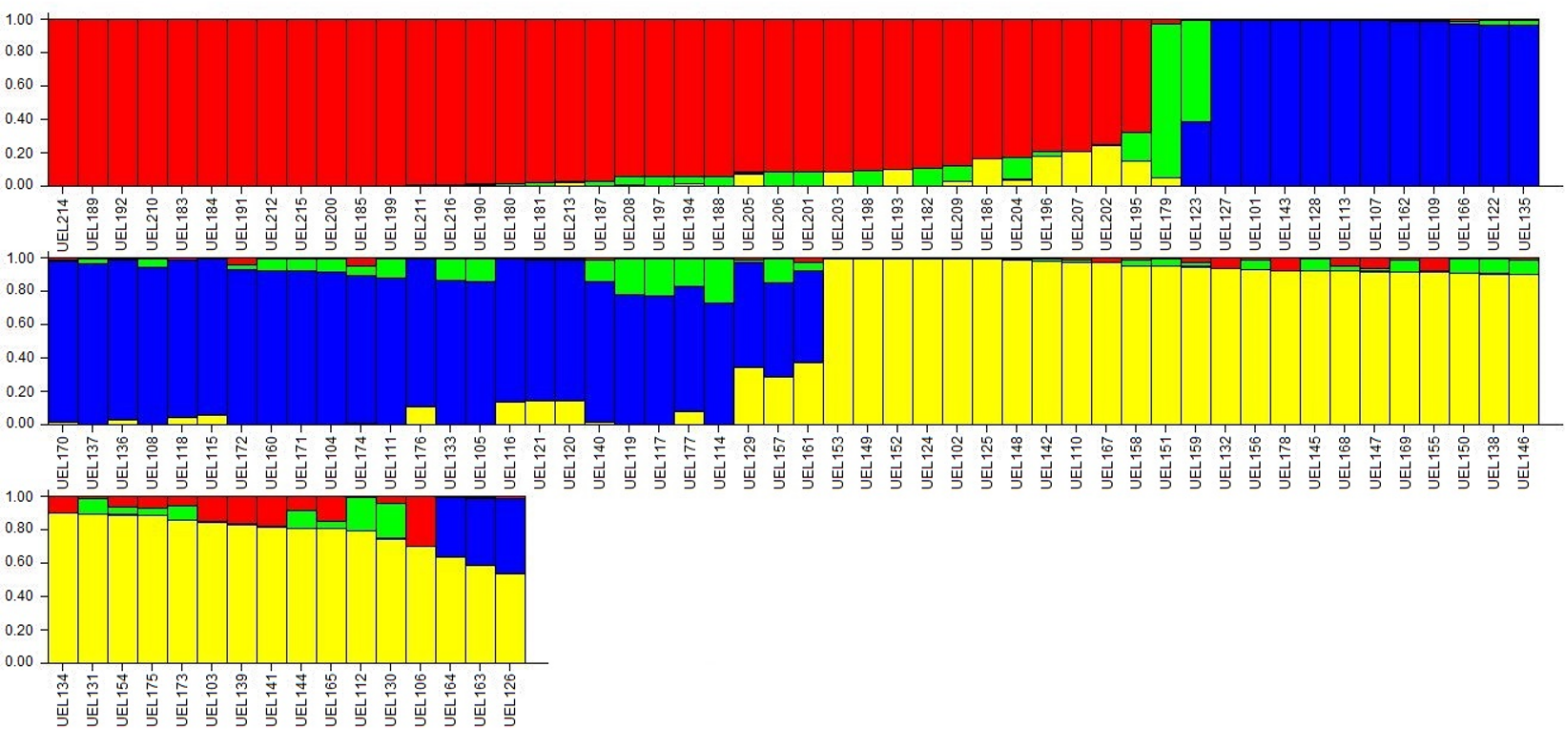
Assignment (K = 4) of 116 *Capsicum baccatum* accessions by the structure bar plots based on six AFLP combinations of primer. Four colors represent different clusters. The y-axis displays the estimated percentage membership of each accession in a determined cluster.

Based on simulations provided by Structure software and the methodology of the Δk value proposed by Evanno et al. [24], the optimal K was four (Fig 7). An agreement of results of Structure analysis with UPGMA clustering was observed. However, four accessions were classified as admixture for having an adhesion coefficient lower than 0.6 for all groups.

The analysis of molecular variance (AMOVA) of the domesticated accessions (*C*. *baccatum* var. *pendulum*) of the three genebanks detected higher variation within (83.18%) rather than among the collections (16.82%).

## Discussion

### Morphological characterization of fruits

The characterization of fruit morphology by the descriptors proposed by Biodiversity International detected broad variability among the accessions studied. The variability in fruit color and shape and the use of new descriptors, not proposed in the original list (e.g. fruit surface and fruit shape at blossom end), evidenced the enormous phenotypic variability in fruits of C *baccatum* accessions. Some accessions (for example, UEL139 and UEL157) also had more than one color before reaching the mature stage. These results were also observed by Sudré et al. [13], who verified wide variability in color of the *Capsicum* spp. accessions.

The wide phenotypic variability in fruits of species of the genus *Capsicum* was also reported in studies with *C*. *chinense* [12,27], *C*. *annuum* [28], *C*. *frutescens* [29,30], and *C*. *baccatum* [4,8,10]. The morphological variability observed in *C*. *baccatum* fruits may be related to the wide geographic distribution of the species in diverse climatic and environmental conditions, which enables the selection of genotypes more adapted to local conditions [7,31].

Our results demonstrate that some descriptors were essential for the distinction between groups, especially fruit shape, in agreement with the study of Baba et al. [12], which also mentioned this descriptor as essential for the distinction of *C*. *chinense* accessions. Some traits related directly to fruit shape were also essential for the separation of the groups.

The characterization and quantification of the phenotypic variability of the fruits are also highly relevant with a view to their conservation and use in plant breeding programs. The accessions of *C*. *baccatum* with longer and elongated fruits, which are predominant in groups G3 and G5 (e.g. UEL103, UEL107, UEL111, UEL113, UEL122, UEL129, UEL135, UEL136, UEL151, UEL156, UEL174, UEL175, UEL182, UEL205, and UEL214), can be included in breeding programs of red chili pepper (Fig 1). The red chili pepper is highly appreciated in Brazil, particularly in the South and Southeast, and is generally consumed fresh, in sauces or in the form of dehydrated flakes [11,32].

Large and long fruits [33], are generally more attractive for the fresh pepper market in Brazil, while smaller fruits with a higher dry mass content are more suitable for the dehydrated food industry. Another important trait is the wall thickness, since fruits with a thicker wall are more resistant against damage during post-harvest handling and have a fresher appearance than fruits with a thinner wall.

The accessions with campanulate fruits, predominantly allocated in group G2 (UEL115, UEL120, UEL121, UEL125, UEL127, UEL145, UEL149, UEL149, UEL152, UEL153, UEL159, UEL168, UEL169, UEL181, UEL188, UEL191, UEL211, and UEL213), represent the *cambuci* or bishop’s hat pepper. This pepper, native to Brazil, is characterized by the peculiarly "bell-shaped" fruit, has little or no pungency, and is widely appreciated in Brazilian cuisine [11].

Other accessions, such as UEL105, UEL119, UEL123, UEL146, UEL158, UEL198, and UEL201, have small fruits with different colors during maturation. The attractive and aesthetic value of ornamental peppers is related to the color change during fruit maturation, as well as the different fruit shapes and sizes [34]. In Brazil, the market of ornamental peppers is extensive and therefore an alternative for small rural producers [14,35].

The differentiation of the groups formed by Ward-MLM was confirmed by the analysis of canonical variables and Mahalanobis’distance. This statistical procedure, which allows the combined analysis of quantitative and qualitative data, consists of two stages. In the first stage, the groups are defined by analysis with Ward's clustering algorithm and a Gower dissimilarity matrix [21], and in the second, the data are grouped by the Modified Location Model (MLM) analysis [19]. This methodology has been used to analyze morphoagronomic traits in several studies, e.g., for common bean [36], tomato [37] and *Capsicum* spp. [12,13,38]. The evaluation of 56 *Capsicum* spp accessions using the Ward-MLM procedure, for the analyses of 26 (15 qualitative and 11 and quantitative) descriptors, Sudré et al. [13] stated that allowed a separation of the species *C*. *annuum*, *C*. *frutescens*, *C*. *baccatum*, and *C*. *chinense*, confirming the importance of a simultaneous use of morphological and agronomic traits [13].

### Molecular characterization

The high polymorphism generated by six AFLP primer combinations clearly shows that these markers were highly efficient to identify polymorphisms among accessions of *C*. *baccatum*. Similar results were found in analyses of the species *C*. *baccatum* and *C*. *anuumm* by Krishnamurthy et al. [39], in which the authors found a percentage of 93.96% polymorphic AFLP markers. In an evaluation of 226 accessions of a *C*. *baccatum* collection from different regions of South America [4], the percentages of polymorphism were 60% and 67% for accessions of *C*. *baccatum* var. *baccatum* and *C*. *baccatum* var. *pendulum*, respectively. These data strengthen the importance of genetic studies analyzing the collections maintained in genebanks.

The UPGMA analysis of the AFLP data separated the accessions in three large groups (Fig 6), in agreement with the results of the Bayesian analysis (Fig 7). In the latter, accession UEL123 was grouped close to UEL179, although considered an admixture, suggesting a genetic proximity among this accessions. The accession UEL157 has ornamental features and due to its proximity to UEL179 may present interesting properties that can be explored in the improvement of ornamental varieties. Besides, the accession UEL179 belongs to the wild variety of *C*. *baccatum* (*C*. *baccatum* var. *praetermissum*). The discrimination between domesticated and wild accessions of *C*. *baccatum* was also observed in another study [4], in which the authors mentioned that the separation of *C*. *baccatum* var. *praetermissum* is much more evident than that of other *C*. *baccatum* varieties, such as the wild variety *C*. *baccatum* var. *baccatum*. There are controversies in the scientific community regarding the taxonomic classification of *C*. *baccatum* var. *praetermissum*. Previous studies, reported by different authors [40,41,42] classified *C*. *baccatum* var. *praetermissum* as *C*. *praetermissum*, a distinct species from *C*. *baccatum*. This classification was supported by the studies of Moscone et al. [41] and Ibiza et al. [42]. On the other hand, in another study [43], it was classified as a variety of *C*. *baccatum* (*C*. *baccatum* var. *praetermissum*). In our results, the position of UEL179 in the dendrogram and in the Bayesian graphics provided evidences in favor of the treatment of *C*. *baccatum* var. *praetermissum* as a taxonomic entity, i.e. *C*. *praetermissum*. However, since our analysis included mostly accessions from Brazil (eastern distribution), additional studies, including more representatives from the entire distribution range (eastern and western) of *C*. *baccatum*, are needed to support this suggestion.

The accessions clustered in each of the groups generated by AFLP markers indicated high genetic variability, whereas no relation between the morphological fruit descriptors and the geographical origin was identified. The absence of an association between geographical origin and molecular markers is most likely due to seed exchange between farmers and the unrestricted fruit transport between different regions of Brazil [12]. This result was corroborated by other studies on *C*. *chinense* [12,44] and *C*. *annuum* [45].

In a genetic diversity study of *C*. *baccatum* accessions [4], two large groups, separated according to the geographical origins (East and West), were identified. The eastern group corresponded to accessions native to Brazil and to eastern Argentina and Paraguay, whereas the western group contained accessions from Peru, Colombia, Chile, Bolivia, and western Argentina. It was observed that the genetic pool of these two areas is homogeneous and that this separation probably resulted from isolation by distance. Therefore, the authors suggest that the species *C*. *baccatum* had different origins of domestication, evolving in two main lines (eastern and western). In our study, almost all accessions were from the eastern region, which may explain the absence of relationships between the genetic data and geographic origin.

The lack of associations between the morphological and molecular data of the fruits indicates that the two analyses are complementary and both necessary for a reliable characterization of a genebank. Several studies with *Capsicum* spp. [10,12,46,47] highlighted the relevance of phenotypic and molecular characterization for an improved understanding of the variability. The combined analysis of morphoagronomic and molecular data provides important findings about the genetic basis of the analyzed accessions, evidencing the great potential of the collection as a source of genes of interest. Our findings can be used to improve the efficiency of conservation and management procedures of the *C*. *baccatum* genebank.

## Conclusions

Wide genetic variability among *C*. *baccatum* accessions was detected by fruit traits and AFLP molecular markers, indicating the high potential of these accessions in pepper breeding programs.

## Supporting information

S1 File Data of morphological fruit descriptors. (XLSX)

S2 File Data of AFLP markers. (XLSX)
